# ZYX-1/Zyxin plays a minor role in oocyte transit through the spermatheca in *C. elegans*

**DOI:** 10.17912/micropub.biology.000489

**Published:** 2021-10-21

**Authors:** Perla G. Castaneda, Nan Wu, Zhongqiang Qiu, Myeongwoo Lee, Erin J. Cram

**Affiliations:** 1 Northeastern University, Department of Biology, Boston, MA, USA; 2 Northeastern University, Department of Bioengineering, Boston, MA, USA; 3 Baylor University, Department of Biology, Waco, TX, USA

## Abstract

In *C. elegans*, oocytes are ovulated into the spermatheca, where they are fertilized before being pushed into the uterus. Contraction in the *C. elegans* spermatheca is driven by circumferential acto-myosin fibers. The *C. elegans* zyxin homolog, *zyx-1*, is expressed in the body wall muscle, pharynx and spermatheca. To our surprise, a CRISPR-generated *zyx-1* deletion allele results in no overt developmental phenotypes, and the spermathecal actin cytoskeleton appears wild type, however, oocyte transit through the spermatheca is slower than in wild type animals. This suggests ZYX-1/Zyxin may regulate spermathecal contraction magnitude or timing of spermathecal bag contraction and/or spermathecal-uterine valve dilation.

**Figure 1. ZYX-1 regulates embryo transit through the spermatheca:**
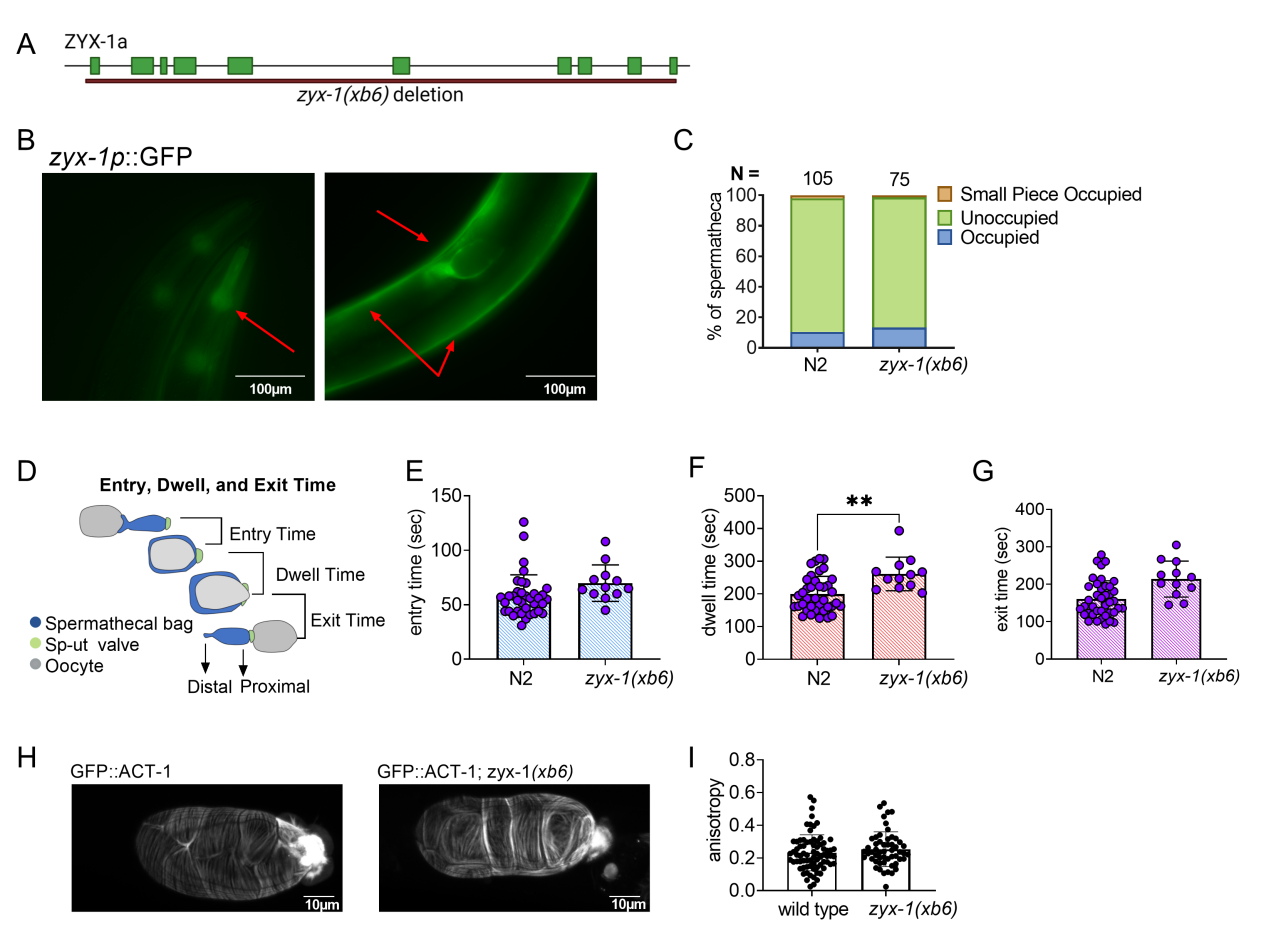
(A) Schematic of ZYX-1a, with location of *zyx-1(xb6)* deletion indicated. Image made with BioRender. (B) Expression pattern for ZYX-1. ZYX-1::GFP is expressed in the pharynx, body wall muscles, and spermatheca. (C) Population assay of wild type animals and *zyx-1(xb6)*. Spermathecae were scored for the presence or absence of an embryo (occupied or unoccupied), or presence of a fragment of an embryo (small piece occupied). The total number of unoccupied spermatheca were compared to the sum of all other phenotypes using the Fisher’s exact t-test. N is the total number of spermathecae counted. (D) Schematic representation of a spermatheca undergoing an ovulation, with entry, dwell, and exit times indicated. Entry time (E), dwell time (F), and exit time (G) analysis of wild type animals and *zyx-1(xb6)* ovulations were compared using Fishers exact t-test. (H) Confocal images of wild type and *zyx-1(xb6)* worms expressing ACT-1::GFP were analyzed using Fibriltool (I) and compared using Fishers exact t-test. Stars designate statistical significance (** p<0.01).

## Description

Zyxin is a LIN-11, Isl1, and MEC-3 (LIM)-domain protein that, in mammalian cells, binds alpha actinin and other cytoskeletal-associated proteins. Zyxin localizes to the integrin-based adhesive structures known as focal adhesions and to stress fibers, which are contractile acto-myosin bundles that regulate cell shape and contractility (Wang *et al.* 2019). The *C. elegans* protein ZYX-1, a protein with similarity to zyxin, migfilin, TRIP6, and LPP (Lecroisey *et al.* 2013), was identified in a screen for binding partners of germline RNA helicase (GLH) proteins (P. Smith *et al.* 2002). Similar to mammalian zyxin, ZYX-1 contains three LIM domains, a potential nuclear export signal (NES) and three proline rich regions (PRR). ZYX-1 is localized to the dense body/Z-disk in body wall muscle and interacts with DEB-1/vinculin (Lecroisey *et al.* 2013), ATN-1/α-actinin (Lecroisey *et al.* 2013) , and DYC-1, a dense-body specific protein that potentially interacts with DYS-1/dystrophin (Lecroisey *et al.* 2008). ZYX-1 might play a role in signaling between the cytoplasm and the nucleus, as a portion of the ZYX-1 protein is translocated from the cytoplasm to the nucleus (Lecroisey *et al.* 2013). Mounting evidence in mammalian cells suggests that zyxin is mechanosensitive and re-localizes to stress fibers that are under tension (Wang *et al.* 2019; M. Smith *et al.* 2010; Schiller *et al.* 2011), and participates in the repair of damaged actin fibers (M. Smith *et al.* 2010). It is not known whether ZYX-1 fulfills a similar function in *C. elegans*.

The *C. elegans* spermatheca is a contractile tube of smooth muscle-like cells found in the hermaphroditic reproductive system. Oocytes are ovulated into the spermatheca, where they are fertilized. The spermatheca then contracts to push the fertilized embryos out through the spermathecal-uterine (sp-ut) valve and into the uterus (Laband *et al.* 2018; Yamamoto, Kosinski, and Greenstein 2006). Oocyte entry stretches the cells of the spermatheca and leads to the formation of basal acto-myosin bundles. These stress fiber-like bundles drive spermathecal contractility (Wirshing and Cram 2017). However, while the spermatheca expresses integin and vinculin, dense bodies are not apparent in this tissue (Ono, Yu, and Ono 2007) and how basal acto-myosin bundles are anchored in this tissue is not well understood. Because of the strain placed on these fibers by embryo transit, we asked if the *C. elegans* homolog of zyxin, ZYX-1, might play a role in assembly or maintenance of the actin cytoskeleton in the spermatheca.

In this study, we investigated the role of *C. elegans* ZYX-1/Zyxin in spermathecal contractility and regulation of the actin cytoskeleton using a novel CRISPR-generated deletion allele. The animals are superficially wildtype and do not exhibit overt cytoskeletal defects. However, when *zyx-1* is disrupted, oocyte transit through the spermatheca is slower than in wild type animals. This suggests ZYX-1/Zyxin may regulate the magnitude or timing of spermathecal contractility.

To characterize the function of ZYX-1/Zyxin in *C. elegans*, we deleted *zyx-1* using CRISPR genome editing. The resulting deletion allele, *zyx-1(xb6)*,is a 13,503 bp deletion with a 12 bp insertion, which removes the entire coding sequence of F24G4.3a (Fig. 1A). The *zyx-1(xb6)* animals were backcrossed four times to N2 to remove off-target mutations, and the deletion was verified by PCR and sequencing. The deletion animals did not exhibit any overt developmental, movement, or growth phenotypes.

We next investigated the expression pattern of ZYX-1/Zyxin using BC14184, a strain expressing a transcriptional fusion of ZYX-1::GFP. ZYX-1::GFP is expressed in the pharynx, body wall muscles, and spermatheca of *C. elegans* (Fig. 1B). Because ZYX-1 is strongly expressed in the spermatheca, we asked if ZYX-1 regulates embryo transits through the spermatheca. Age matched, young adult *zyx-1(xb6)* and N2 animals were scored for spermathecal occupancy using differential interference contrast (DIC) microscopy. No statistically significant increase in spermathecal occupancy was observed (Fig. 1C). While a small piece of an oocyte was observed in the *zyx-1(xb6)* worms, this was also observed in our N2 control.

To detect subtle defects in spermathecal transits, we used video microscopy to record ovulations and transits of *zyx-1(xb6)* and N2 animals and calculated the entry time, dwell time and exit time. Entry time is the time from the opening of the distal spermatheca to the complete entry of the oocyte and closure of the distal spermathecal neck. Dwell time is the time when the embryo is completely enclosed by the spermatheca. Exit time is the time from the opening of the sp-ut valve to the complete expulsion of the embryo into the uterus (Fig. 1D). We observed little difference between the two genotypes in terms of entry time and exit time, but the dwell time was significantly longer in the *zyx-1* deletion than in the wild type (Fig. 1E-G). This suggests that ZYX-1/Zyxin promotes embryo transit through the spermatheca.

In cell culture systems, zyxin is required to maintain stress fibers (M. Smith *et al.* 2010), especially under tension. We speculated that defects in the actin cytoskeleton might explain the observed *zxy-1(xb6)* transit phenotype. To determine if ZYX-1/Zyxin regulates spermathecal actin, we crossed *zxy-1(xb6)* into a strain expressing GFP::ACT-1 and observed spermathecal transits using confocal microscopy. However, no difference was observed in the actin fiber alignment in wild type and *zyx-1(xb6)* spermathecae (Fig. 1H, I).

Here, we demonstrate that ZYX-1/Zyxin is not required per se for spermathecal transits or for actin alignment in the spermatheca. ZYX-1/Zyxin does seem to be involved in regulation of the magnitude or timing of spermathecal contractility in *C. elegans*, however, the mechanism of this regulation remains elusive.

Why might *zyx-1(xb6)* have such mild phenotypes? ZYX-1/Zyxin may only become necessary when the animals are stressed by loss of another regulator. For example, depletion of DYS-1/Dystrophin and ZYX-1/Zyxin in body wall muscle (Lecroisey *et al.* 2008) leads to defects in the actin cytoskeleton and paralysis. Although ZYX-1 is the sole zyxin/migfilin/TRIP6/LPP (Lecroisey *et al.* 2013) homolog in *C. elegans*, other actin associated proteins may serve functionally redundant roles in regulation of the actin cytoskeleton and spermathecal contractility. Future proteomic and/or genetic studies may lead to a better understanding of the role ZYX-1/Zyxin in *C. elegans*.

## Methods

*C. elegans* strains and culture

*C. elegans* were maintained at 23°C, on NGM agar plates ((0.107 M NaCl, 0.25% wt/vol Peptone, 1.7% wt/vol BD Bacto‐Agar, 2.5 mM KPO4, 0.5% Nystatin, 0.1 mM CaCl2, 0.1 mM MgSO4, 0.5% wt/vol cholesterol) seeded with *E. coli* OP50. *C. elegans* BC14184 (ZYX-1::GFP) and N2 strains were obtained from the *C. elegans* Genetics Center.

To delete the RGD motif in *zyx-1* locus in chromosome II, we identified two effective CRISPR sites to delete about 10 kb from exon 1 to 10 from the CRISPR guide RNA Selection Tool, http://genome.sfu.ca/crispr/search.html. According to the intended mutation, 84-mer repair DNA templates containing 25-base homology arms (*zyx-1* null, see the reagent section) were designed and custom-made by IDT Inc., Coralville, IA. Then, the mixture of template DNA (ZYX-1 template name), two crRNA (zyx1start and zyx1stop, see the reagent section), tracrRNA (catalog no. 1072532), and Alt-R Cas9 (catalog no. 1081058) proteins are annealed at 37^o^C and micro-injected into the syncytial gonad arms of N2 animals (P0) with *dpy-10* crRNA as a co-CRISPR marker (Paix *et al.* 2015; Dickinson and Goldstein 2016). The F1 offspring of P0 worms are selected by Dpy and subjected to PCR genotyping to identify worms carrying the desired deletions. Once the F1 mutants are isolated, F2 progeny are screened to identify the homozygous alleles as described below.

PCR was used to verify the genomic modification. Briefly, single animals were lysate in 6 μl worm lysis buffer (10 mM Tris HCl pH 8.3, 50 mM KCl, 2.5 mM MgCl2, 0.45% Tween 20, 0.45% Triton X100) and PK (Proteinase K) and frozen at -80℃. After protease digestion at at 60℃ for 1 hr and denaturation at 95℃ for 15 min, the lysate was used for PCR with GoTaq green master mix using the primer pairs, ZYX-1 null Forward (TGTCTTTCAGCTTGGGTCGT) and ZYX-1 null Reverse (GGTTGGCATCCGTACTCGAA) to amplify the *zyx-1* locus. The edit was verified by sequencing. Isolated PCR products were sequenced to confirm the mutations. The established homozygous *zyx-1* deletion lines were backcrossed to N2 several times to reduce off-target effects. The resulting strain, UN2013 (*zyx-1(xb6)*), was crossed with UN1502 (GFP::ACT-1) to produce UN2016 (Y66H1Bp::GFP::ACT-1; *zyx-1(xb6)*).

Preparation of populations of young adult hermaphrodites

To obtain age-matched young adult hermaphrodites, dauer nematodes were placed on fresh, seeded NGM plates and raised for 48 hours. Eggs were harvested from gravid adults using alkaline lysis as described (Hope 1999), and were left to starve overnight to arrest at L1. L1 animals were raised in a 23°C incubator and observed after 50-54 hours.

DIC, fluorescence and confocal microscopy

Animals were mounted on 5% agarose in 0.1 M sodium azide and observed immediately with DIC microscopy to score gonad phenotypes. Imaging was performed on a Nikon Eclipse 80i microscope with a 60× oil-immersion lens using SPOT Advanced software (Version 5.3.5) and a charge-coupled device camera. Frames were captured at a rate of 1 Hz. Image stacks were reassembled and analyzed using ImageJ software (Schindelin *et al.* 2012). Fluorescence microscopy was performed on a Nikon Eclipse 80i microscope equipped for epifluorescence. Images were captured with a SPOT RT3 CCD camera using SPOT Advanced software (Diagnostic Instruments; Sterling Heights, MI, USA). Confocal microscopy of animals expressing GFP::ACT-1 was performed on a Zeiss LSM 710 confocal using Zen software and a Plan-Apochromat 63x/1.40 oil objective lens using a 488-nm laser (Kelley *et al.* 2020).

Quantification with ImageJ

We used ImageJ software (version 2.1.0/1.53c) to analyze the ovulation movies and to measure the length of the worms. Open ImageJ, right-click on the ‘straight line’ tool, select ‘freehand line’, draw a line along the shape of the worms, and press command+M to get the pixel value. The length of worms is calculated from the pixel value through a conversion based on the calibrated scale bar.

Analysis of actin alignment with Fibriltool, an ImageJ plugin

Fibriltool was used essentially as described in Boudaoud, A *et al.*, (Boudaoud *et al.* 2014) Briefly, the steps are to open the file, split the channels, apply z project, and process and save the image in PNG format (a key step for success), before following the steps in the article.

Statistics

All of the statistical analysis was performed using GraphPad Prism (version 9.2.0). For comparing transit timepoints and anisotropy, the Student’s t-test was used. The Fisher’s Exact test was used to compare proportions of occupied spermatheca.

## Reagents

**Table:** 

Oligo Name	Sequence (5’-3’)
ZYX1NULLF (PCR genotyping)	TGTCTTTCAGCTTGGGTCGT
ZYX1NULLR (PCR genotyping)	GGTTGGCATCCGTACTCGAA
ZYX1WTR (PCR genotyping)	ATCCCTGAGCTTTTGGGGGT
zyx-1 null REPAIR TEMPLATE (84 bases)	ggggggaatggaaattgttgactgatggctcgcTTGCTAGCGCTAGCtagcggatgccgagtctggaatagtgctgaaggag
zyx1start (crRNA)	GGGGTCCCATagatcagtag
zyx1stop (crRNA)	tgactgatggctcgcTTACG

**Table:** 

Strain	Genotype	Available from
N2	*Caenorhabditis elegans*	CGC
UN1502	*xbls1502[Y66H1Bp::GFP::ACT-1, pRF4(rol-6(su1006))]*	Cram Lab
UN2013	*zyx-1(xb6)*	Cram Lab
UN2016	*zyx-1(xb6);* *xbls1502[Y66H1Bp::GFP::ACT-1, pRF4(rol-6(su1006))]*	Cram Lab
BC14184	*sEx14184[rCes F42G4.3b::GFP + pCeh361]*	CGC
